# Comparing Localization Performance of IEEE 802.11p and LTE-V V2I Communications

**DOI:** 10.3390/s21062031

**Published:** 2021-03-13

**Authors:** Rreze Halili, Maarten Weyn, Rafael Berkvens

**Affiliations:** IDLab-Faculty of Applied Engineering, University of Antwerp-imec, Sint-Pietersvliet 7, 2000 Antwerp, Belgium; rreze.halili@uantwerpen.be (R.H.); maarten.weyn@uantwerpen.be (M.W.)

**Keywords:** IEEE 802.11p, LTE, CRLB, GDOP, vehicle localization accuracy

## Abstract

The future of transportation systems is going towards autonomous and assisted driving, aiming to reach full automation. There is huge focus on communication technologies expected to offer vehicular application services, of which most are location-based services. This paper provides a study on localization accuracy limits using vehicle-to-infrastructure communication channels provided by IEEE 802.11p and LTE-V, considering two different vehicular network designs. Real data measurements obtained on our highway testbed are used to model and simulate propagation channels, the position of base stations, and the route followed by the vehicle. Cramer–Rao lower bound, geometric dilution of precision, and least square error for time difference of arrival localization technique are investigated. Based on our analyses and findings, LTE-V outperforms IEEE 802.11p. However, it is apparent that providing larger signal bandwidth dedicated to localization, with network sites positioned at both sides of the highway, and considering the geometry between vehicle and network sites, improve vehicle localization accuracy.

## 1. Introduction

Wireless communication technologies are all around our daily life, used by smartphones, smart homes, and smart cities, making our days easier and more productive. These technologies are being expanded and integrated into vehicles and road infrastructure, targeting the reduction of road accidents, enhancement of road safety, reliability, and traffic management.

Over the recent years, the emphasis on intelligent vehicle research has turned to Cooperative Intelligent Transport Systems (C-ITS) which enables information exchange between vehicles (vehicle-to-vehicle, V2V) and between vehicle and transport infrastructure (vehicle-to-infrastructure, V2I), referred as vehicle-to-everything (V2X) communication [1]. The concept and development of such systems have been in the focus of industry, academia, standard institutions, and highway administration institutions [2], aiming to support road navigation, vehicles tracking, monitoring, emergency services, access to traffic conditions, weather conditions, air pollution, etc. However, despite many vehicle-related location-aware applications, localization or positioning accuracy remains the main problem [2]. In addition to this, according to the literature review, vehicular networking technologies are being designed for communication and not for localization purposes. However, the system should make available sub-meter-level localization accuracy to comply with the demands of the automotive sector [3].

Traditionally, Global Navigation Satellite System (GNSS) receivers have been used for vehicle localization. However, a standalone GNSS receiver and its improved systems known as Differential GNSS (DGNSS) and Real-Time Kinematic (RTK) have their inaccuracy in location information in dense and indoor environments, such as tunnels, urban canyons, and dense forests [4,5]. As an alternative solution to this, it is seen the explosion of potential localization capabilities using technologies such as ad-hoc IEEE 802.11p and cellular LTE-V.

IEEE has released IEEE 802.11p known as WAVE in US and ETSI ITS-G5 in Europe, for vehicular ad-hoc networks (VANETs). Wi-Fi-based V2X technology includes categories such as vehicle-to-vehicle (V2V) communications and vehicle-to-infrastructure (V2I) communications [4,6]. IEEE 802.11p uses orthogonal frequency-division multiplexing (OFDM) for both uplink and downlink. The entire signal is wideband (i.e., occupies a 10 MHz channel) [7].

3GPP has released Release 14 of Long-Term Evolution (LTE) referred as LTE-V2X or LTE-V. Cellular-based V2X technology includes communication between vehicle-to-vehicle (V2V), vehicle-to-pedestrian (V2P), vehicle-to-infrastructure (V2I), and vehicle to network (V2N) [6]. It uses single-carrier frequency-division multiple access (SC-FDMA) for uplink and OFDM for downlink. The channel bandwidth can be 10 and 20 MHz [8].

Both technologies, ad-hoc IEEE 802.11p and cellular LTE-V are considered to be complementary to each other [7,9], while they have also been topic for different performance comparison studies, considering delay, reliability, quality of service, data rate, mobility, coverage, economic size, etc. [7,8,10,11,12]. However, there is a lack of studies about the capability of these technologies to localize vehicles using communication signals. This because of limited access on network operator deployments, lack of positioning signals provided by mobile operators, non-synchronized and small number of required base stations.

In [13,14] are presented positioning results by using LTE downlink signals collected in a portable experimental setup during a car drive in the town of Rapperswil, Switzerland. The study analyzes the received signal by combining time, frequency, spatial, and cell ID domains for position tracking. In general, an error of 59.83 m with a coverage of 72% has been obtained using a simple Channel Impulse Response-based timing algorithm with NLOS rejection, whereas error an of 48.47 m with full coverage has been obtained using a Threshold-to-noise ratio-based estimator. The study is performed using a limited number of base stations of mobile operators and concludes the need for exploiting different cell sizes to improve localization.

Hence, we conduct a comparative study and investigate the achievable localization accuracy of the IEEE 802.11p and LTE-V, using vehicle-to-infrastructure signals. V2X air interfaces use the Uu interface, which connects users’ equipment or onboard units (OBUs) with road-side units (RSUs) or eNBs, which have the role of base stations. Real data measurements in an actual testbed are incorporated as a prominent feature to have preliminary results of these signals’ behavior and their capabilities to be used for an optimized vehicular network. 3GPP LTE path-loss model [15] is chosen over the WINNER [16] to model the signal propagation. Furthermore, the study is done by providing numerical results through simulation of the two aforementioned technologies on two different vehicular network design scenarios, with the same propagation conditions and same frequency band.

We analyze the Cramer–Rao Lower Bound (CRLB) for Time Difference of Arrival (TDOA) measurements in an additive white Gaussian noise (AWGN) channel, with a zero mean and a variance dependent on the geometry between vehicles and base stations. The role of this geometrical distribution of base stations included in the estimation is further examined using Geometric Dilution of Precision (GDOP). Two different vehicular network design scenarios related to the placement of base stations in use are simulated. In addition to this, the Least square (LS) algorithm is considered to obtain the TDOA technique performance on vehicular localization. The LS algorithm uses ranges of IEEE 802.11p and LTE-V to calculate the vehicle position location.

The main contribution of this study is the comparison results on maximum achievable localization accuracy provided by IEEE 802.11p and LTE-V using V2I downlink communication signals. Another novel contribution is this work is also the development of a comparison model regarding localization accuracy limits, obtained by V2I communication signals. This model can be adapted by other vehicular network designs and other frequency bands, where is a need to predict, examine, and improve route infrastructure deployment, technology signal coverage, frequency band, propagation conditions, and vehicular network poor design. In our work, this model is used to analyze and develop the testbed over the E313 highway in Antwerp, Belgium.

The remainder of this paper is divided as follows: Section 2 is a brief overview of related works. Vehicular network designs, measurements setup, derivation and simulation of CRLB, GDOP, and LS algorithm are presented in Section 3. Simulation results, discussions, and comparisons between two technologies, two vehicular network designs, and the relation between used parameters are presented in Section 4. Conclusions are summarized in Section 5.

## 2. Related Work

Different research is done on the achievable localization accuracy by using LTE positioning reference signal (PRS) and Cell ID signal. Time of Arrival (TOA) estimation algorithm is performed on the LTE downlink signals carried on measurements from multiple base stations [14,17]. The study in [18] considers the performance limits of the V2I ranging-based localization technique using LTE networks. TOA of the cell-specific reference signals (CRS) of LTE belonging to different mobile operators is considered in [19,20] to increase positioning coverage. Observed time difference of arrival (OTDOA) technique in Rel 14 LTE networks [21], using dedicated positioning reference signals in a simulation model is performed in [22]. Other Rel 14 LTE positioning protocol, primary and secondary synchronization signals transmitted by base stations are used for navigating in [13,23,24]. Although 3GPP Rel-16 or millimeter-wave 5G specification provides a new radio for positioning by assuming sub-meter level accuracy for 95% of the service areas and vehicular cases [25]. Position estimation through millimeter-wave using Multiple-input and Multiple-output (MIMO) in 5G systems are examined in [26,27,28], by focusing on advantages of the MIMO system, the large number of antennas, large bandwidths, high carrier frequencies, cooperative localization, and new design of services coverage, smaller cells, and dense networks. This release is expected to inherit the positioning mechanisms of previous standards and adopt potential new methods [3]. Some improved algorithms using TOA technique are also found in VANETs localization using IEEE 802.11p [2,3]. Most of these results are only experimental results using signals emulated in laboratories or Software Defined Radios.

Localization performance accuracy is evaluated with the theoretical localization limits defined by the CRLB. Most of the CRLB studies [3,17,29,30,31,32,33] consider a constant variance that does not depend on the estimated parameter. However, in practice, the input variance in the covariance matrix depends on the geometry between transmitters and receivers. Therefore, in this study, we have computed the standard deviation of the TDOA measurements as a function of the transmitter positions. This results in a more realistic value for CRLB.

To scale the impact of the geometry between transmitters and receivers, the GDOP parameter is considered. This criterion is used for choosing appropriate base stations in cellular communication systems [34]. The first published paper that presents the GDOP of hyperbolic multilateration systems is found in [35]. In [34] the GDOP is used to select appropriate base stations in cellular communication systems while performing TDOA. Then [36] used GDOP to optimize the deployment of the base stations for range measurement positioning systems by using a sided regular polygon with one base station in the center of the polygon, which resulted in decrease the value of DOP. A weighted GDOP (WGDOP), using error statistics property and by selecting specific measurement units is done in [37]. In vehicular networks for hyperbolic TDOA is used in [3,38]. In this work, we have used DOP 2D to compare two different vehicular network designs.

To model the transmission of signals for CRLB estimation and technologies comparison, physical layer performance should be addressed. In this regard, compared with V2V channels, a research gap is mentioned to support V2I communication coverage prediction and interference analysis [39,40,41]. The study in [42] compares IEEE 802.11p and LTE-V technologies considering vehicular communication in an urban non-line of sight (NLOS) scenario. Differences between line of sight (LOS), NLOS due to foliage, and NLOS due to buildings, are shown on the GEMV2 simulated model using the V2I field measurements campaign in 5.9 GHz frequency band performed in the city center of Bologna [40]. Other conducted extensive field testing on the link quality of IEEE 802.11p V2I channels in urban scenarios are found in [43,44].

The study in [36] suggest an empirical path-loss model for V2I channels, which relates path-loss exponent with antenna height, by analyzing the shadowing effect and the large and small-scale fading effect for three different link types (LOS beneath, NLOS, and LOS above the environment). Then, WINNER B1, C2, and D1 models are developed for vehicular networks for urban micro-cell (UMi), typical urban macro-cell (UMa), and rural macro-cell (RMa) scenarios [16]. An adaptation of WINNER channels models is done by Rel 14 by standardizing the 3D Channel Model for LTE [15]. We refer to this adaptation as the 3GPP LTE path-loss model.

## 3. Vehicular Networks Design

### 3.1. Scenarios Definition

To compare two technologies in respect of achievable vehicle localization accuracy and two proposed network designs on the E313 highway testbed, located in Antwerp, Belgium, are performed four different simulated scenarios. These simulations are done to have preliminary results on the achievable vehicle localization accuracy and to choose which of these two network designs should be considered for deployment on the E313 highway.

**Figure 1:**
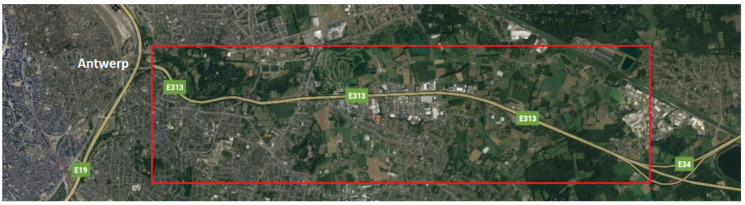
The segment of the two-way E313 highway in Antwerp, Belgium. © 2020 Google.

The E313 highway testbed consists of interconnected hardware including vehicle part named onboard unit (OBU), base stations or network sites part named road-side units (RSUs) planned to be placed along the highway, backbone and testbed management software platform, and optical fiber ring along the highway. The considered segment is presented in Figure 1.

#### 3.1.1. RSUs Location

In this study, for both technologies, IEEE 802.11p and LTE-V, we consider the case when RSUs are deployed at both sides of the highway as the first proposed vehicular network design, and the case when RSUs are deployed at one side of the highway as the second proposed vehicular network design. The planned location coordinates for all RSUs depend on the route infrastructure or gantries placed on the highway. Thus, the inter-distance between RSUs in the study is not constant, it varies depending on the placement of the highway gantries. This inter-site distance has an impact on localization limitations.

**Figure 2:**
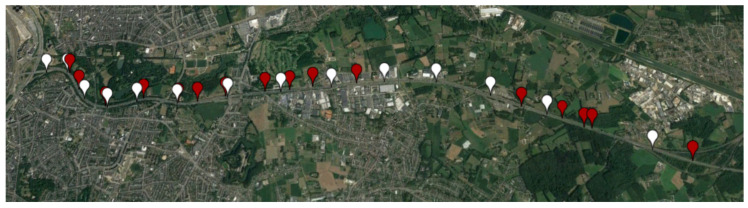
Map of the environment of the simulated area where RSUs are placed on two sides of the E313 highway. On one side are white color marked sites, and on the other side of the E313 highway are placed the red color marked sites. © 2020 Google.

In Figure 2 are shown RSUs placed periodically at two sides of the highway. The white color marked sites are at the first side of the highway and the red color marked sites are placed at the other side of the highway. We have considered all two-sided 30 RSUs for the first technologies comparison and one-sided 15 RSUs for the left comparison.

#### 3.1.2. Time Difference of Arrival

TDOA determines the location of the vehicle by evaluating the difference in arrival times of the communication signals from separated network sites. As in the previous study [45], to comply with the TDOA technique, the network sites are synchronized to a reference clock, and the vehicle has its own clock. Since there is no need to have synchronization between network sites and the vehicle, these assumptions are correct.

We consider a single OBU placed at $x_{k}={\left[x_{k},y_{k}\right]}^{T}$ at $t_{k}$ time. Meantime, we assume that the positions of RSUs are presented as $s_{k}^{i}={\left[x_{k}^{i},y_{k}^{i}\right]}^{T}$, where i=1,2,3…M, depending on the number of network sites. For every location, while driving, OBU measures the times of arrival of the downlink signals received from the RSUs and multiplies it with the speed of light to have the distances between the OBU and RSUs. The Euclidean distance between the OBU and RSU is computed as

(1) $$\begin{array}{c} r^{i}=\sqrt{{\left(x-x^{i}\right)}^{2}+{\left(y-y^{i}\right)}^{2}}. \end{array}$$

Considering the above equation, we obtain distances from each RSUs. Then, differences between every RSUs distances with one reference RSU distance are used to estimate the two-dimensional position of the OBU. The reference RSU, or reference network site is the most powerful RSU, the one located on the shortest distance from the location of OBU.

(2) $$\begin{array}{c} z_{1j,k}=h_{1j,k}\left(x_{k}\right)+u_{1j,k}. \end{array}$$

Referring the expression on Equation (2), the resulted range differences $z_{1i,k}$ are sum of exact range differences $h_{1j,k}$ and the additive white Gaussian noise AWGN with a standard deviation $\sigma_{i}$, non-constant variance $\sigma_{1j}^{2}=\sigma_{1}^{2}+\sigma_{j}^{2}$, and joint conditional Gaussian distribution covariance matrix defied below, Equation (3), where j=2,3…M.

(3) $$R=\left(\begin{array}{ccc} \sigma_{1}^{2}+\sigma_{2}^{2} & \ldots & \sigma_{1}^{2}\\ \vdots & \ddots & \vdots \\ \sigma_{1}^{2} & \ldots & \sigma_{1}^{2}+\sigma_{M}^{2} \end{array}\right).$$

Following the TDOA technique, OBU needs to have communication with at least four RSUs, to perform localization. For scenarios considered in simulation, OBU communicates with all RSUs which are placed in a defined range distance. This maximum coverage distance of each RSU is approximated considering the technology real data measurements performed on the E313 highway. While moving, OBU will change its reference RSU depending on the level of received signal strength and time of arrival. This network design avoids signal interference of time and frequency resources.

#### 3.1.3. Transmit Power and Frequency Bandwidth

Vehicular communication between RSUs placed along the highway and the OBU placed in the vehicle is based on multicarrier orthogonal frequency-division multiplexing (OFDM) modulations. Therefore, both technologies use the same Modulation and Coding Scheme.

The antenna of each network site is formed by a uniform linear array of $M_{a}$ elements with known orientation, while the OBU antenna is omnidirectional.

For IEEE 802.11p simulation, it is considered OFDM physical layer signal with a bandwidth of 10 MHz, 64 sub-carries (each has 156.25 kHz), the maximum transmit power is 23 dBm, and its received sensitivity level defined in the corresponding standard in [46] is −85 dBm. Although for LTE-V we have 600 sub-carries (each has 15 kHz) and according to the LTE standard [21] the maximum transmit power is 46 dBm, its received sensitivity level is −90.4 dBm [47]. However, sensitivity levels achieved by commercial devices or prototypes can be −92 dBm for IEEE 802.11p [48] and −103.5 dBm for LTE-V [49].

In this work, we have considered the maximum transmit power that technologies can provide to compare their best performance on achievable localization accuracy.

Other parameters required for simulation are summarized in Table 1 and are further explained and used in Section 3.2.

#### 3.1.4. Propagation Conditions

We analyze the downlink signals from RSUs or emitters to one OBU, through additive white Gaussian noise (AWGN). These transmitted signals are subject of LOS and NLOS propagation modes caused by the surrounding environment between RSUs and OBU, while the latter one is driving on the highway. Propagation modes are defied using the LOS/NLOS probability which is determined by the distance between RSUs and OBU.

The surrounding environment is characterized by the influence of traffic jams, trees, and vegetation. Also, various infrastructural highway metal components have a considered impact. No high buildings or objects were identified. To consider the impact of this environment we compare two propagation models with real measurements tests obtained on the E313 highway testbed.

Since deterministic models are considered not computationally efficient, because of their complexity and divergence on different environments, we apply the WINNER propagation model [50] and the 3GPP LTE model [51].

Also, here we add the shadow fading defined as the variability in the received power due to signal obstructions. This parameter is accurately modeled as a random Gaussian variable with a standard deviation.

Further explanation about the appropriate propagation channel model used in this work is provided below in Section 3.3.

### 3.2. Cramer Row Lower Bound for TDOA

The accuracy of the OBU two-dimensional position estimation using TDOA depends on the transmitted signals from RSUs to OBU. Since these signals or waveforms are subject to random phenomena such as noise, fading, shadowing, multipath, non-line of sight propagations, and interference [17,39,52], the estimated position is impacted by uncertainty. To scale this uncertainty and predict the achievable accuracy level is used root-mean-square error (RMSE) of the position estimate (x). Another way is to have Cramer–Rao Lower Bound (CRLB).

The position error in m is,

(4) $$ex=\sqrt{E[∥x-x{∥}^{2}]}⩾\sqrt{tr(CRLB(x))},$$

where *E* is the expected value and tr is trace operator. CRLB is the lower bound on the variance of any unbiased estimator [53]. This time we use to determine the lower bound of the achievable positioning accuracy. This lower bound can be used to determine the network design guidelines for accurate vehicle localization [3]. CRLB is computed from the inverse of the Fisher Information Matrix (FIM) *J* defined in [53]. Considering the derivation of the CRLB in an AWGN channel done in [53], the CRLB in m, is expressed as

(5) $$CRLB\left(x\right)={\left(D^{T}R^{-1}D\right)}^{-1},$$

where

(6) $$D=\left(\begin{array}{ccc} \frac{x-x_{1}}{d_{1}}-\frac{x-x_{2}}{d_{2}} & \ldots & \frac{y-y_{1}}{d_{1}}-\frac{y-y_{2}}{d_{2}}\\ \vdots & \ddots & \vdots \\ \frac{x-x_{1}}{d_{1}}-\frac{x-x_{M}}{d_{M}} & \ldots & \frac{y-y_{1}}{d_{1}}-\frac{y-y_{M}}{d_{M}} \end{array}\right),$$

and *R* is defined in Equation (3).

CRLB for TOA and TDOA is studied in different scenarios in [3,5,17,29,30,33]. In [17,29,33] the covariance matrix R does not depend on the vehicle position, which results in an approximated constant covariance matrix. However, in this study, we include the position of the vehicle in the covariance matrix, so no constant variance for different measurements is obtained. To model this covariance matrix for both technologies IEEE 802.11p and LTE-V, standard deviation and variance of the TDOA should be defined. According to [3,17] and the explanations above, the variance of a TDOA measurement is the sum of the associated TOA measurements. This parameter is defined as shown in the expression below and depends on frequency *f* (in Hz), relative power weight ${p_{k}}^{2}$ of sub-carrier *k*, number of sub-carriers *N*, sub-carrier spacing $F_{sc}$ (in Hz), signal bandwidth *B* (in Hz), and signal to noise ratio $SNR_{j}$,

(7) $$\sigma_{j,TOA}^{2}=\frac{c^{2}}{8\pi^{2}M_{a}N_{m}SNR_{j}\frac{F_{sc}^{2}}{N}\sum_{k}p^{2}k^{2}}.$$

$N_{m}$ is the total number of measurements and $M_{a}$ is the number of antenna elements. In (7) $SNR_{j}$ is defined in dB as,

(8) $$\;SNR_{j}=P_{max}-SF_{j}-N-L_{j}+147-10log\left(NF\right)-10log\left(B\right),$$

(9) $$\;L_{j}=max\left(PL\left(d_{j}\right)-G_{t}-G_{r},MCL\right),$$

where $P_{max}$ (in dBm) is the maximum transmit power, $SF_{j}$ (in dB) is the shadow fading, NF (in Hz) is receiver noise figure, *B* (in Hz) is signal bandwidth, $G_{t}$ and $G_{r}$ (in dB) are transmitter and receiver antenna gains, and MCL (in dB) is the minimum coupling loss between OBU and RSU. $PL(d_{j})$ (in dB) are macroscopic losses defined by propagation path-loss models for vehicular communication.

### 3.3. Measurements and Channel Models for Vehicular Communication

To have a realistic simulated estimation of the CRLB, there is a need to model the signal propagation channel very accurately. To validate this model real data measurements are performed, hence we could obtain the behavior characteristics of the signal propagation on the selected part of the E313 highway.

**Figure 3:**
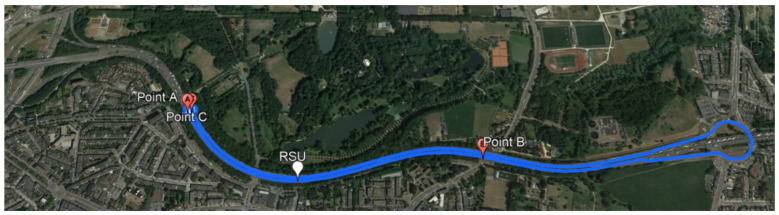
Map of the environment of conducted field measurements, while driving on the E13 highway. The OBU starts its journey on point A, toward point B, and then back to point C. © 2020 Google.

The first measurements were conducted on the link quality and radio wave propagation of the IEEE 802.11p V2I channels in one of the E313 highway segments (Smart Highway: https://www.fed4fire.eu/testbeds/smart-highway/, (accessed on 1 February 2021)). where we were provided with one RSU and one OBU. The measurements were performed on a 5.2 km route, driving on the E313 highway from point A toward point B, and then back to point C, Figure 3.

**Figure 4:**
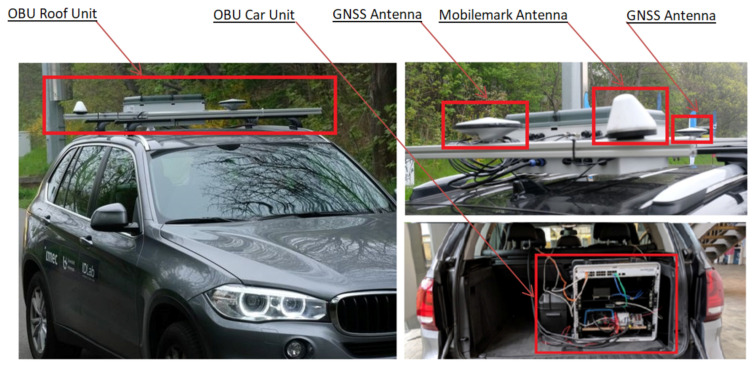
The OBU placed on the vehicle. The OBU Roof Unit mounted on the roof (**left**) together with the antennas (**right**) and the OBU Car Unit placed inside the vehicle (**right**).

As shown in Figure 4, one part of the OBU is placed 1.8 m high on the vehicle roof and the other part is placed inside of the vehicle. OBU contains the vehicle part of the ITS-G5 Cohda Wireless containing Cohda MK5 OBU, one Mobilemark MGW-303 antenna, two 5.9 GHz antennas, and one active GNSS antenna.

It also contains a GNSS module AsteRx-m2a with RTK correction, and GNSS PolaNt-x MF antennas. Inside the vehicle is the car unit which contains the processing unit with an independent power system, which can power the OBU for several h.

**Figure 5:**
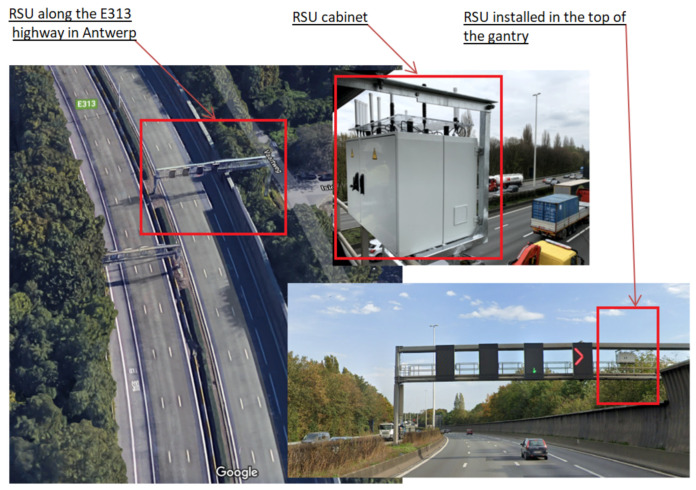
The RSU placed on the E313 highway. The location of the considered gantry (**left**) and the RSU cabinet (**right**) together with its location installation (**right**). © 2020 Google.

The deployed RSU is placed on one of the gantries of the E313 highway as shown in Figure 5. Inside this module is a large electrical cabinet that contains different technologies and a processing unit. In our interest are the ITS-G5 Cohda Wireless MK05 RSU, two antenna connectors connected to 5.9 GHz antennas, internal GNSS receiver (connected to a GNSS antenna mounted inside the RSU), GNSS AsteRx-m2 OEM Base, together with a GNSS PolaNt-x MF antenna.

The effective transmitted power of ITS-G5 IEEE 802.11p module was 23 dBm. The position of the vehicle was observed by the very accurate GNSS receiver used also for device synchronization. For every received packet on this communication, we obtained the received signal strength (RSS) in dBm, speed of the vehicle, its location, signal to noise ratio, and time.

The obtained measurements tests results are used to evaluate and compare the WINNER propagation model and the 3GPP LTE model in the aspect of propagation prediction performance. The considered models are developed for different areas including urban micro-cell (UMi), typical urban macro-cell (UMa), and rural macro-cell (RMa) scenarios.

WINNER propagation model noted as D1 represents radio propagation in largely rural areas with low building density. The height of the transmitted antenna is typically much higher than the average building height. The receiver antenna is supposed to be located inside of a building or a vehicle with a velocity between 0 to 200 km/h [50].

The 3GPP LTE path-loss model can be applied in the frequency range between 2–6 GHz, for antenna heights higher than the average building height, and receiver antenna height between 1 m to 10 m. It considers street width and building density and heights. Both models consider the transition between LOS and NLOS propagation conditions [51].

### 3.4. Dilution of Precision

As mentioned above, the expected placement of all RSUs in the testbed depends on the gentries’ location on the E313 highway. This placement, respectively the RSUs and OBU geometric relationship does have a huge impact on the estimated positioning accuracy. Thus, we investigate the Horizontal DOP (HDOP) values for two simulated scenarios where RSUs placement and density are different.

DOP is a unit-less number and represents the geometric effect of base stations and the mobile station on the relationship between measurement error and positioning determination error. It is a function of geographical positions of used base stations [54]. This unit-less value is used to identify highway parts where we have poor RSUs placements and to select the most appropriate set of RSUs in the calculation, which gives the minimum positioning error.

In addition to this, we are interested to see if there is a relation between the obtained values of HDOP, CRLB, and TDOA estimation.

As for the CRLB estimation, we have selected the same reference RSU, which is the most powerful RSU for each OBU location. Then, the geometry matrix *D* defined in Equation (6) is used to calculate the HDOP as shown in Equation (10),

(10) $$\;HDOP\left(x\right)=\sqrt{tr{\left(D^{T}D\right)}^{-1}}.$$

### 3.5. Least Square Method for TDOA

To continue our comparison between two technologies and two network designs in the study, we also use the least square (LS) method [53] to estimate the TDOA OBU location. This is done using the calculated pseudo-ranges for hyperbolic TDOA, Equations (11)–(13). This algorithm is used to have ranges of IEEE 802.11p and LTE-V, calculate the position, and compare it with the real position or location of the OBU on the E313 highway.

(11) $$E=\left(\begin{array}{cc} \;x_{1}-x_{2} & y_{1}-y_{2}\\ \vdots & \vdots \\ \;x_{1}-x_{M} & y_{1}-y_{M} \end{array}\right)$$

(12) $$F=\frac{1}{2}\left(\begin{array}{c} \;x_{1}^{2}-x_{2}^{2}+y_{1}^{2}-y_{2}^{2}-r_{21}^{2}-r_{21}d_{1}\\ \vdots \\ \;x_{1}^{2}-x_{M}^{2}+y_{1}^{2}-y_{M}^{2}-r_{M1}^{2}-r_{M1}d_{1} \end{array}\right)$$

(13) $$\;LS_{TDOA}={\left(E^{T}E\right)}^{-1}E^{T}F$$

LS algorithm is estimated considering signal propagation modeling and range differences explained above. Each simulated TOA measurement error is assumed to be uniformly distributed over zero mean and variance which depends on RSUs and OBU geometric position as shown in Equation (7).

## 4. Results and Discussions

This section shows numerical evaluations of the IEEE 802.11p and LTE-V technologies capabilities for accurate vehicle localization, using V2I communication. Results of real data measurements are used to model propagation path losses caused by the surrounding environment around one segment of the E313 highway, in Antwerp, Belgium.

The V2I network consists of RSUs periodically located with certain inter-site distances, road-side separation, and an OBU placed on the vehicle, which drives on the defined lane. All other parameters are the same as presented in Table 1. Therefore, the comparison is done between the two above mentioned technologies capabilities IEEE 802.11p and LTE-V, considering two different vehicular network designs.

### 4.1. Results from Real Data Measurements

**Figure 6:**
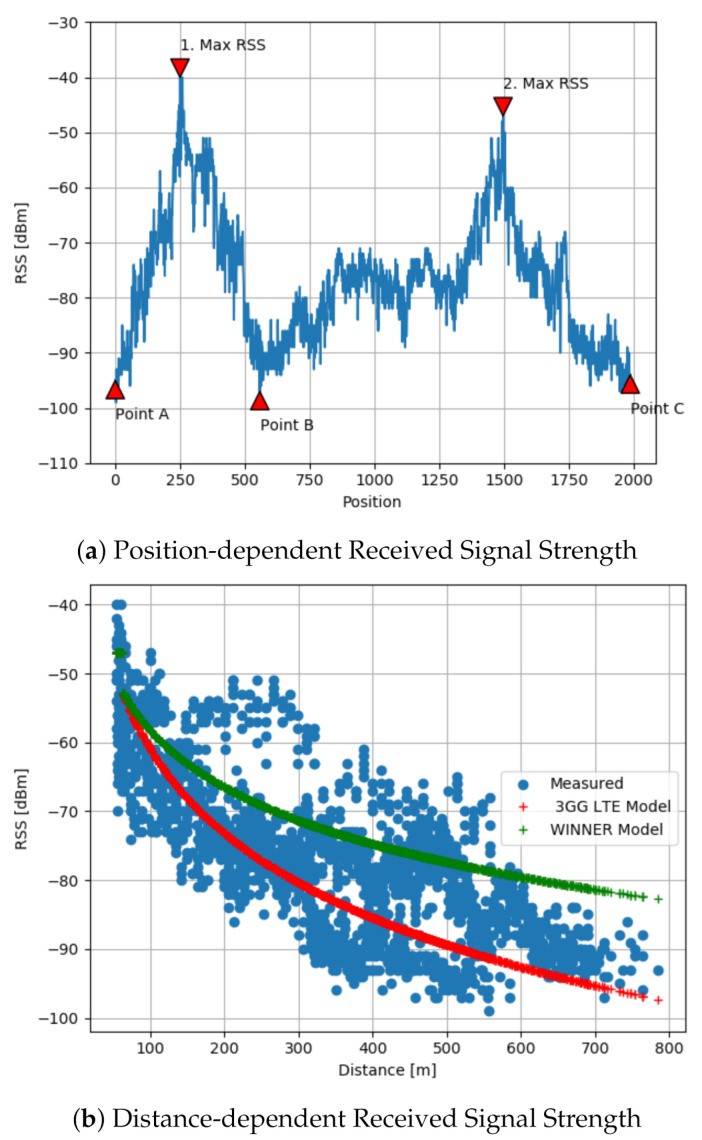
(**a**) Position-dependent Received Signal Strength using ITS-G5, IEEE 802.11p at 5.9 GHz frequency. (**b**) Received Signal Strength at 5.9 GHz frequency as a function of TX-RX separation distance. The blue circles are values obtained from the E313 highway measurement campaign. The red and green crosses represent the resulted values form the 3GPP LTE Model, respectively WINNER Model.

While driving on the highway shown in Figure 3, OBU and RSU were communicating on LOS and NLOS modes. In Figure 6a is shown the corresponding received signal strength (RSS) depending on the position of the moving vehicle or OBU. When OBU approaches the pointed RSU, from point A in the direction of point B, we have a higher level of RSS expressed in dBm. The OBU receives the highest RSS level pointed in Figure 6a as 1. Max RSS. While heading forward, the RSS values are lower after the OBU crosses the RSU and continues its drive. The minimum value of the RSS is −99 dBm, recorded at a large distance between the sender and receiver (near point B). After point B, when the RSU signal coverage is not available, the OBU stops receiving communication, until the OBU receives the RSU signal again on the return drive toward point C.

To model and explain this behavior the WINNER path-loss model [16] and its adaptation on the 3GPP LTE model [15] are used. To measure the performance of the propagation models in terms of accurate derivation of the path-loss, respectively the RSS values, we use Mean Absolute Error (MAE), which presents the differences between the real RSS obtained on field measurements and modeled or estimated RSS. The MAE performance metric shows that the 3GPP LTE model performs better than the WINNER model. This is also shown in Figure 6b. Thus, we use the 3GPP LTE to model the signal propagation, expected path-loss, and RSS on the highway.

### 4.2. Results from Simulated Measurements

The positioning accuracy simulation findings show that there is a difference between two technologies and between two vehicular network designs. The first details are presented in Table 2.

**Figure 7:**
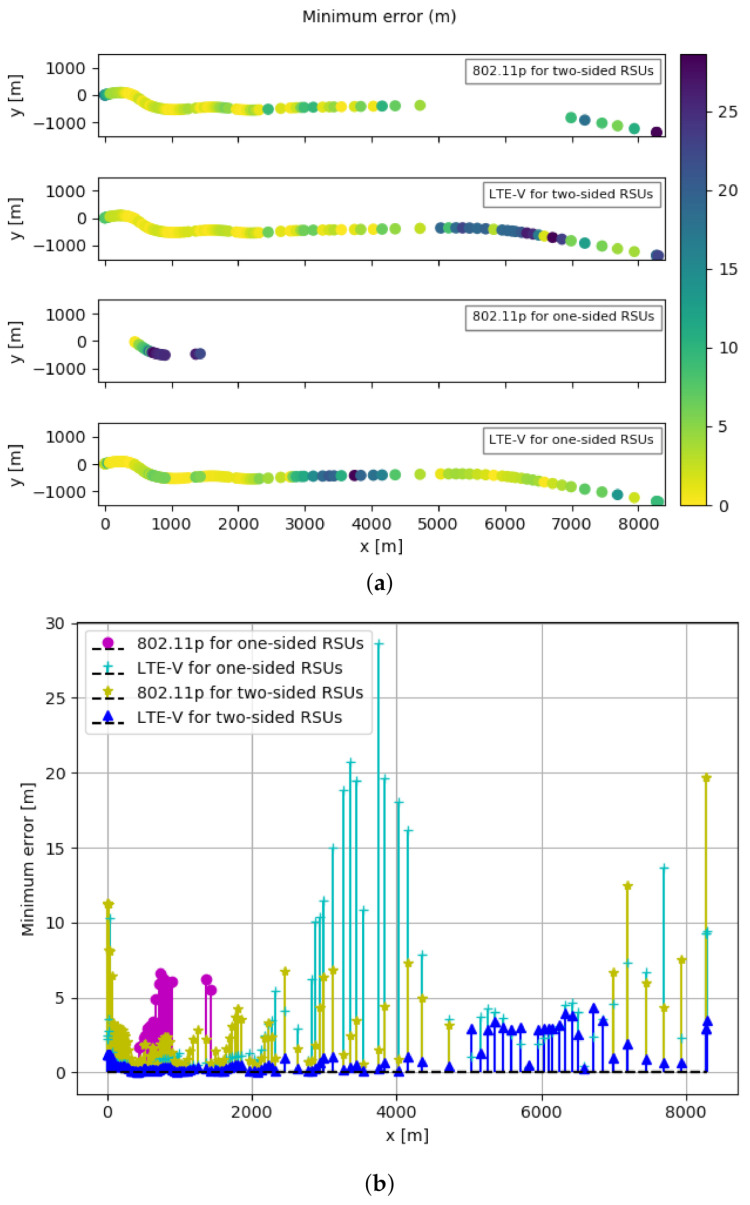
Localization performance comparison using minimum position error for two-sided and one-sided RSUs for IEEE 802.11p and LTE-V. (**a**) Minimum position error maps. (**b**) Minimum position error values.

The minimum achievable errors obtained using CRLB for IEEE 802.11p and LTE-V technology when RSUs are placed at both sides of the highway and when RSUs are placed at one side of the highway are shown in Figure 7a,b. In Figure 7a are shown results obtained on the movement of the OBU on the highway on xy-plane. These xy-plane values are a transform of the geodetic coordinates specified by latitudes, longitudes, and height to the local north-east-down Cartesian coordinates specified by xNorth, yEast, and zDown.

When RSUs are placed at both sides of the highway as shown in Figure 7a, the minimum errors we obtain for IEEE 802.11p are between 0.37 m to 19.67 m, while for LTE-V are 0.06 m to 4.33 m. Furthermore, when RSUs are placed at one side of the highway, the values for minimum errors vary between 1.66 m to 6.65 m for IEEE 802.11p, and between 0.17 m to 28.63 m for LTE-V. This comparison is more visible in Figure 7b, where we show the numerical result of minimum achievable errors on the y axis.

Despite minimum, maximum, and average error values, in Table 2 is also the coverage column. This parameter is directly impacted by the different maximum transmitted power that two technologies can provide (23 dBm by 802.11p compared to 46 dBm by LTE-V). The coverage percentage shows the number of locations or cases where TDOA CRLB values are obtained. In the case of two-sided RSUs at all included vehicle locations, for IEEE 802.11p and LTE-V, we obtain CRLB for 82.46%, respectively 100% of cases or locations. On the other hand, obtaining CRLB for IEEE 802.11p and LTE-V for one-sided RSUs is possible for just 16.67%, respectively 100% of vehicle locations. In Figure 7a the coverage aspect is noticed by the missing points in the map or missing values in the graph in Figure 7b.

In addition to this, achieving sub-meter level accuracy is possible for 25.44% for IEEE 802.11p, respectively 73.68% of locations for LTE-V, when having RSUs on two sides of the highway. However, for one-sided RSUs this value is 0% for IEEE 802.11p and 46.49% for LTE-V.

Vehicle positions or locations where we could not obtain values for minimum achievable errors are because the vehicle could not reach communication with four RSUs to have TDOA or the covariance showed in Equation (3) is a singular matrix. The first limitation can be solved if we add more RSUs on the highway. Previous study [3] suggests having inter-side distances set to 200 m and road-side separations to 15 m for rural macro-cell to ensure at least three LOS RSUs for 95% of the cases. The effect of the second limitation is reduced when ignoring the measurement noise, which results from the OBU location. Studies as [17,29] consider a constant covariance matrix and independent of the vehicle position.

As a comparison done between IEEE 802.11p and LTE-V, we see that the minimum localization error achieved using IEEE 802.11p is higher than LTE-V when RSUs are placed on both sides of the highway. We have higher values when OBU uses LTE-V on one-sided RSUs; however, the coverage of IEEE 802.11p is just 16%. This implies that better performance compared to four cases is achieved by LTE-V when two-sided RSUs are taken into consideration.

While analyzing the above results, it is noticeable that they have different values; however, they have the same tendency of changes. This implies that despite technology, bandwidth, number of sub-carriers, transmitted power, and coverage, a very important factor is the geometry which stands between OBU and RSUs. To scale this impact factor, we use 2D GDOP or HDOP, which shows the strong importance that RSUs placement has on the OBU localization. Following the explanations in the simulation section related to HDOP, the findings are shown in Table 3.

**Figure 8:**
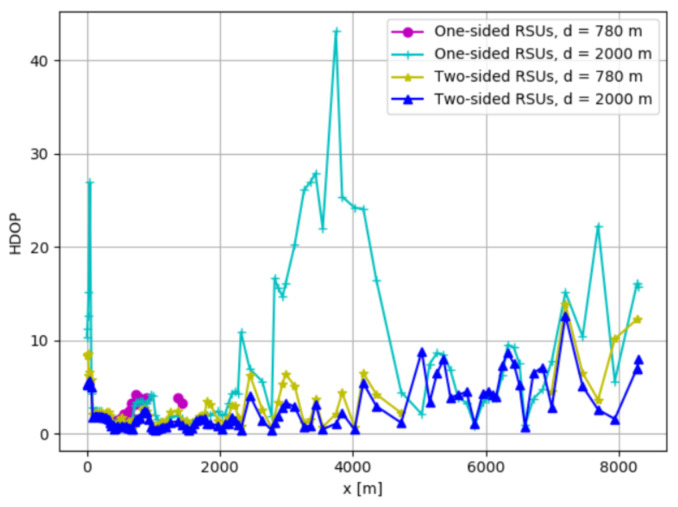
Localization performance comparison using HDOP for two-sided and one-sided RSUs.

The comparison is done considering the coverage range or limit and the placement of RSUs along the highway, as shown in Figure 8. Furthermore, despite standard comparison parameters which include minimum, maximum, average, and coverage, here we have HDOP values that are below 2 and indicate very good geometry between RSUs and OBU. Then, average performance is achieved when HDOP sides between 2–5, and when HDOP sides above 5, we have bad localization performance.

Results show that the minimum HDOP is 0.37, found for a vehicle location when two-sided RSUs are used in a 2000 m coverage range, and the maximum HDOP value is 43.07 using one-sided RSUs in the same coverage limit. When we have the same coverage range d = 2000 m, it is important to point out that the number of location cases where we could obtain HDOP or coverage rate is 100%, while all other parameters minimum, maximum, and intervals on the table indicate that we have lower HDOP when using two-sided RSUs compared to one-sided RSUs. The same can be said for d = 780 m too; however, as shown in the figure, the number of location cases where we could obtain HDOP for one-sided RSUs is very small compared to two-sided RSUs, 82.46% compared to 16.67%.

These results indicate that two-sided RSUs deployments have better position than one-sided RSUs. In rural areas due to the higher distances between the OBU and RSUs, the levels of HDOP are higher and this approach provides a reduced improvement of localization accuracy [3]. In addition to this, having a specific RSUs placement can ensure lower HDOP using fewer RSUs [34,38].

Simulations are conducted to evaluate the performance of the proposed Least Square Method in TDOA-based location algorithms. Table 4 shows the comparison between IEEE 802.11p and LTE-V for two network designs in the study. As we can see, the error values for the IEEE 802.11p implemented on both sides of the highway varies between 0.42 m to 71.25 m. Its average location error is 7.50 m, while it ensures coverage for 82.46% of cases, where 6.14% of them are less than 1 m.

LTE-V implemented on both sides of the highway varies between 0.02 m to 24.72 m, the average error is 2.20 m, its coverage is just 100% of locations, and 43.86% of these locations errors values are below 1 m. Following the changes in the number of RSUs on the network design, the LS for IEEE 802.11p varies between 2.03 m and 13.91 m, its average error is 6.22 m, while 0% of locations in the study are less than 1 m. Its coverage is 16.67%. When LTE-V is simulated on one side RSUs of the highway, its values are between 0.05 m to 30.51 m, its average is 2.90 m, while its coverage includes 100% of cases, and 43.86% of them are lower than 1 m.

**Figure 9:**
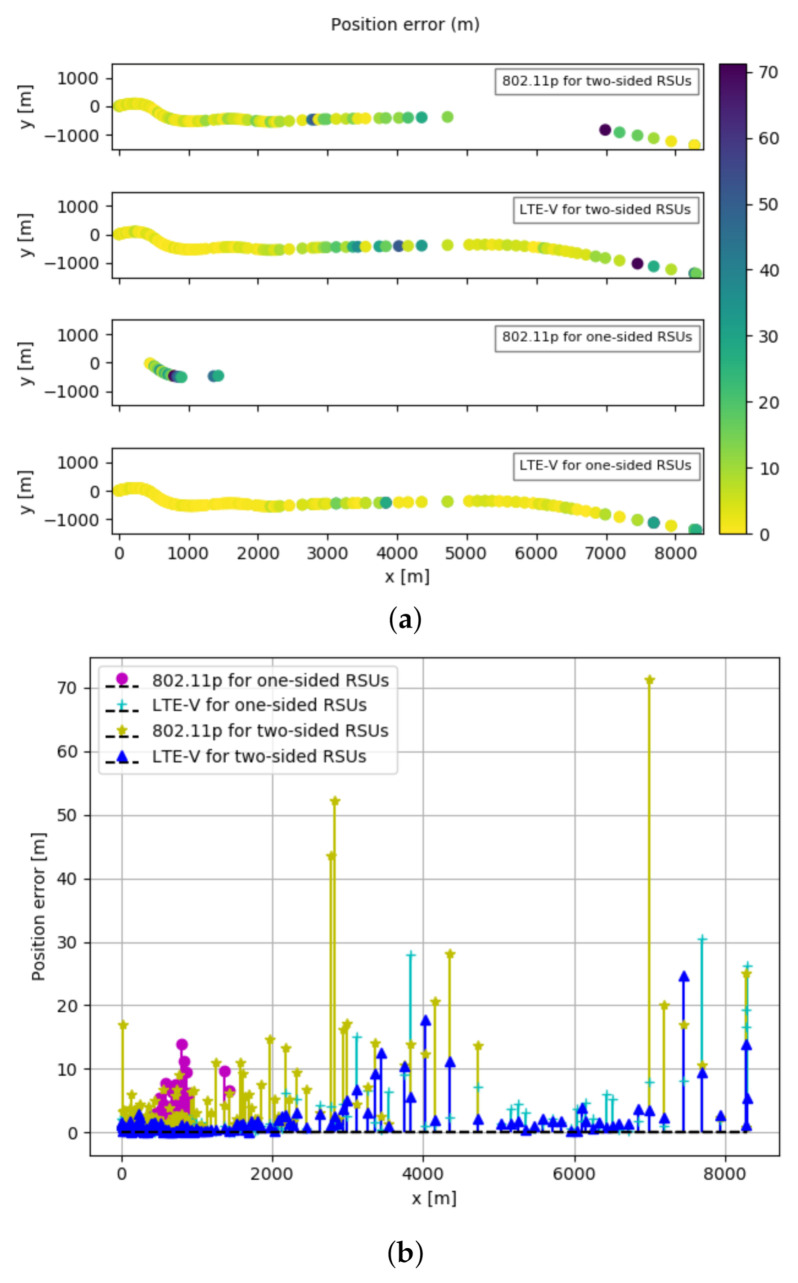
Localization performance comparison using Least square error for two-sided and one-sided RSUs and technologies IEEE 802.11p and LTE-V. (**a**) Position error maps. (**b**) Position error values.

Comparing the third parameter, LS algorithm shows again that LTE-V outperforms IEEE 802.11p for two scenarios in the study. As we can see the LTE-V error values when it is implemented on both sides of the highway, or on one side of the highway, are lower than the IEEE 802.11p. In addition to this, the number of sub-meter error values obtained from the LTE-V is much higher than the IEEE 802.11p. Figure 9a,b is applied to show another way of noticing these differences when the OBU travels on the E313 highway.

**Figure 10:**
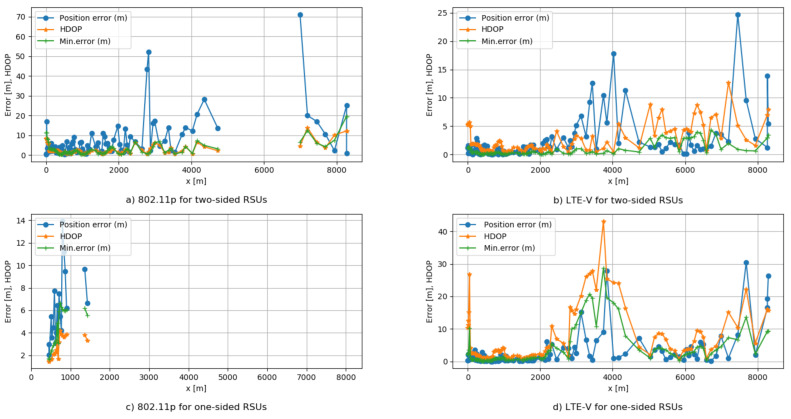
Comparison of minimum position error, HDOP, and position error values of (**a**) IEEE 802.11p for two-sided RSUs, (**b**) LTE-V for two-sided RSUs, (**c**) IEEE 802.11p one-sided RSUs, (**d**) LTE-V for one-sided RSUs.

Figure 10 is applied to examine if there is any relationship between the minimum possible error derived by CRLB, HDOP, and LS algorithm for error estimation. We consider in greater depth results that give more information to our observations. It is important to stress that there is a consistency of the obtained values or similar differences for the same OBU positions and surrounded RSUs in the study.

Although comparing the received parameters in the study, minimum error, HDOP, and position error, for IEEE 802.11p, Figure 10a,c, the findings show that HDOP values have resulted in much lower than the localization error values. This does not happen in the case of LTE-V, shown in Figure 10b,d. Recall here that the number of sub-carriers used for positioning signals considered for LTE-V is much higher than IEEE 802.11p, and the maximum potential transmitted power in LTE-V is 46 dBm, whereas for 802.11p is 23 dBm. This implies the impact that signal bandwidth and transmitted power have in obtaining good localization results.

Moreover, similar differences are also shown for LTE-V, Figure 10b,d. We can notice that higher values of HDOP imply larger localization errors. This shows that in these OBU positions, there is a poor geometry between RSUs and OBU. In such situation, changing the RSUs location can improve the localization accuracy.

In addition to this, the coverage columns of the above parameters contain different values. Whereas for LTE-V for two network designs, we have 100% of OBU positions, for IEEE 802.11p is 82.46% when using two-sided RSUs and only 16.67% of OBU positions when using one-sided RSUs. This is more visible in Figure 10a,c, where we see the missing position locations on the graphs. This fact indicates that all OBU locations where we could not have value for positioning error are because of an RSU absence in the region. In the parts where we cannot obtain values, there is a need to add RSUs, in optimal positions where we could obtain minimum HDOP.

Here it is important to add the fact that the considered locations of RSUs are determined by the current infrastructure on the E313 highway. As a result, the inter-side and number of RSUs are predetermined. In this situation, we have noticed that when performing TDOA using one-sided RSUs placed on a linear way on the E313 highway we have the worst localization accuracy, while when the RSUs were deployed on two sides of the highway and the OBU location was near these RSUs, we received the best result.

While driving on the E313 the RSUs antennas heights is above the road infrastructure and the surrounding buildings which are also low in density. This situation is expected to change when the OBU is found in an urban area where the antenna height of the RSUs and OBU are below the rooftop of the surrounding buildings. In this new situation, the propagation conditions are mainly characterized by non-line-of-sight modes, higher path losses, diffraction, and multipath.

To overcome these obstructions, different urban network vehicular deployment studies consider small cells where we have a small coverage range of RSUs and a higher number of them. In such a scenario IEEE 802.11p and LTE-V should have the same transmit power, which would result in the same coverage range. In this case, the achievable accuracy depends only on the signal bandwidth.

In our first observations for the E313 highway, in a situation where both technologies perform on the same transmit power and coverage range, the LTE performs better because of allocated sub-carriers resources for positioning. Furthermore, LTE-V can perform from 1.4 to 20 MHz bandwidth, including 10MHz, which we have assumed in our scenarios. Besides this, according to [38], LTE-Advanced which uses carrier aggregation (CA) can achieve a system bandwidth up to 100 MHz.

Mitigation of boundaries in the respect of RSUs deployments, analyses of new propagation environments such as urban areas and tunnel environments, mitigation of NLoS measurements, potential uses of reconfigurable meta-surfaces, are upcoming topics to be exploited in the future to enhance achievable localization accuracy.

## 5. Conclusions

Vehicle localization performance comparison between IEEE 802.11p and LTE-V V2I Communications for two different vehicular network designs is performed in this study. Real data measurements are obtained on the E313 highway in Antwerp, Belgium, to model the propagation communication channels in the environment under the study. The propagation results and vehicular network configurations are used to simulate vehicle-to-infrastructure (V2I) communication channels between transmitters referred to as road-side units (RSUs) and receiver referred to as onboard unit (OBU).

Scenarios when RSUs are placed on both sides of the highway and when RSUs are placed on one side of the highway are considered to analyze achievable positing accuracy for two technologies. The propagation path losses are modeled using the 3GPP LTE model, which better fit the real data measurements compared to the WINNER model.

The communication signals used for vehicle localization are received by multiple RSUs using time difference of arrival (TDOA) measurements in an additive white Gaussian noise channel with a zero mean and a distance-dependent variance. Both technologies operate with a 10 MHz bandwidth at 5.9 GHz frequency and have 64 sub-carrier and 300 sub-carriers for IEEE 802.11p and LTE-V, respectively. We have considered their maximum transmitted power, to compare their maximum achievable localization accuracy.

To analyze the potential minimum achievable positioning accuracy is used Cramer–Rao Lower Bound (CRLB), to scale the impact of the geometry between RSUs and OBU, while the last one is driven on a defined lane of the highway is used Horizontal Dilution of Precision (HDOP), and to obtain the positioning error is used Least square (LS) algorithm for TDOA technique.

It is found that using V2I communication signals to have vehicle localization depend on the signal bandwidth, higher bandwidth results in smaller errors. Furthermore, the inter-site distance or distance between RSUs have a huge impact on localization accuracy. Coverage ranges, antennas heights, the infrastructure used, propagation conditions, and surrounding environment, all have an impact on the localization performance and its limits.

Investigating the maximum of lower achievable positioning error shows that LTE-V has lower values than IEEE 802.11p, 4.33 m compared to 19.67 m. When using two-sided RSU, on the simulated testbed, 73.68% of error values in LTE-V are less than 1 m, while for IEEE 802.11p, are 25.44%. When using one-sided RSUs, LTE-V obtained value is 28.63 m compared to IEEE 802.11p, 6.65 m. However, the positioning availability is just 16.66% for IEEE 802.11p compared to 100% for LTE-V, same the sub-meter accuracy cases are 0.0% compared to 49.49% for LTE-V. When obtaining HDOP, the positions where we could obtain OBU location using two-sided RSUs, HDOP values are lower compared with the cases when we use one-sided RSUs.

Our work shows that LTE-V deployed on two-sided RSUs performed better than IEEE 802.11p according to LS algorithm too. Maximum resulted error values are 24.72 m for LTE-V and 71.25 m for IEEE 802.11p. Due to its bandwidth in use for localization, LTE-V outperforms IEEE 802.11p.

Simulated results as analytical expressions show a correlation between the obtained localization errors and HDOP, which fact permits a conclusion that HDOP is a good metric to predict the magnitude of a TDOA error and to identify poor geometry of RSUs in a vehicular network.

The results are used to identify where we expect acceptable localization accuracy, where we have high error values, and where we have poor RSUs deployment on the E313 highway, for the planned real data measurements with all RSUs, placed in the testbed. In the future, we plan to compare planned real data measurements with these numerical simulations, include different propagation environments such as urban areas and tunnel environments, mitigation of NLoS measurements, potential uses of reconfigurable meta-surfaces, to contribute on high accurate vehicular positioning. 

## Figures and Tables

**Table 1:** Simulation parameters of the vehicular network scenarios for IEEE 802.11p and LTE-V.

Parameter	IEEE 802.11p	LTE-V
P${}_{max}$	23 dBm	46 dBm
f	5.9 GHz	5.9 GHz
G${}_{t}$	15 dBi	15 dBi
G${}_{r}$	5 dBi	5 dBi
h${}_{t}$	8 m	8 m
h${}_{r}$	1.8 m	1.8 m
B	10 MHz	10 MHz
F${}_{sc}$	156.25 kHz	15 kHz
N	64	600
SF	6 dB and 8 dB	6 dB and 8 dB
N${}_{m}$	1	1
NF	9 dB	9 dB
M${}_{a}$	1	1
MCL	70 dB	70 dB
p${}^{2}\left(n\right)$	1	1
Modulation	OFDM	OFDM

**Table 2:** The minimum, maximum, average, coverage, and accuracy level of minimum achievable error for two-sided and one-sided RSUs, for IEEE 802.11p and LTE-V.

Scenario	Min	Max	Average	Coverage	Sub-Meter
802.11p (two-sided RSUs)	0.37 m	19.67 m	3.66 m	82.46%	25.44%
LTE (two-sided RSUs)	0.06 m	4.33 m	0.87 m	100%	73.68%
802.11p (one-sided RSUs)	1.66 m	6.65 m	4.39 m	16.66%	0.0%
LTE (one-sided RSUs)	0.17 m	28.63 m	3.66 m	100%	46.49%

**Table 3:** The minimum, maximum, average, coverage, and ranges of HDOP for two-sided and one-sided RSUs.

Scenario	Min	Max	Average	Coverage	HDOP < 2	2 < HDOP < 5	HDOP > 5
HDOP (two-sided RSUs, d = 780 m)	0.59	13.88	2.91	82.46%	39.47%	28.95%	14.03%
HDOP (two-sided RSUs, d = 2000 m)	0.37	12.67	2.56	100%	64.04%	17.54%	18.42%
HDOP (one-sided RSUs, d = 780 m)	1.47	4.22	2.84	16.67%	4.39%	12.28%	0.00%
HDOP (one-sided RSUs, d = 2000 m)	0.87	43.07	6.74	100%	35.09%	31.58%	33.33%

**Table 4:** The minimum, maximum, average, coverage, and accuracy level of LS algorithm for two-sided and one-sided RSUs, for IEEE 802.11p and LTE-V.

Scenario	Min	Max	Average	Coverage	Sub-Meter
802.11p (two-sided RSUs)	0.42 m	71.25 m	7.50 m	82.46%	6.14%
LTE (two-sided RSUs)	0.02 m	24.72 m	2.20 m	100%	43.86%
802.11p (one-sided RSUs)	2.03 m	13.91 m	6.22 m	16.67%	0.0%
LTE (one-sided RSUs)	0.05 m	30.51 m	2.90 m	100%	43.86%

## References

[1] ERTRAC (2019). ERTRAC Working Group Connectivity and Automated Driving.

[2] Ansari A.R., Saeed N., Ul Haq M.I., Cho S. (2018). Accurate 3D localization method for public safety applications in vehicular Ad-Hoc networks. IEEE Access.

[3] Del Peral-Rosado J.A., Seco-Granados G., Kim S., López-Salcedo J.A. (2019). Network Design for Accurate Vehicle Localization. IEEE Trans. Vehic. Technol..

[4] Fascista A., Ciccarese G., Coluccia A., Ricci G. (2017). A Localization Algorithm Based on V2I Communications and AOA Estimation. IEEE Signal Process. Lett..

[5] Hossain M.A., Elshafiey I., Al-Sanie A. (2018). High precision vehicle positioning: Towards cooperative driving based on VANET. J. Internet Technol..

[6] Chen S., Hu J., Shi Y., Peng Y., Fang J., Zhao R., Zhao L. (2017). Vehicle-to-Everything (v2x) Services Supported by LTE-Based Systems and 5G. IEEE Commun. Stand. Mag..

[7] Cecchini G., Bazzi A., Masini B.M., Zanella A. Performance comparison between IEEE 802.11p and LTE-V2V in-coverage and out-of-coverage for cooperative awareness. Proceedings of the 2017 IEEE Vehicular Networking Conference (VNC).

[8] Molina-Masegosa R., Gozalvez J. (2017). LTE-V for Sidelink 5G V2X Vehicular Communications: A New 5G Technology for Short-Range Vehicle-to-Everything Communications. IEEE Veh. Technol. Mag..

[9] Dressler F., Kargl F., Ott J., Tonguz O.K., Wischhof L. (2011). Research challenges in intervehicular communication: Lessons of the 2010 Dagstuhl Seminar. IEEE Commun. Mag..

[10] Chen S., Hu J., Shi Y., Zhao L. (2016). LTE-V: A TD-LTE-Based V2X Solution for Future Vehicular Network. IEEE Internet Things J..

[11] Bazzi A., Masini B.M., Zanella A., Thibault I. (2017). On the Performance of IEEE 802.11p and LTE-V2V for the Cooperative Awareness of Connected Vehicles. IEEE Trans. Veh. Technol..

[12] Molina-Masegosa R., Gozalvez J., Sepulcre M. (2020). Comparison of IEEE 802.11p and LTE-V2X: An Evaluation with Periodic and Aperiodic Messages of Constant and Variable Size. IEEE Access.

[13] Driusso M., Babich F., Knutti F., Sabathy M., Marshall C. Estimation and tracking of LTE signals time of arrival in a mobile multipath environment. Proceedings of the 9th International Symposium on Image and Signal Processing and Analysis, ISPA 2015.

[14] Driusso M., Marshall C., Sabathy M., Knutti F., Mathis H., Babich F. (2017). Vehicular Position Tracking Using LTE Signals. IEEE Trans. Veh. Technol..

[15] (2018). Study on 3D Channel Model for LTE. https://portal.3gpp.org/desktopmodules/Specifications/SpecificationDetails.aspx?specificationId=2574.

[16] (2007). IST-WINNER II Project Deliverable D1.1.2, “WINNER II Channel Models”.

[17] Del Peral-Rosado J.A., López-Salcedo J.A., Seco-Granados G., Zanier F., Crisci M. Achievable localization accuracy of the positioning reference signal of 3GPP LTE. Proceedings of the 2012 International Conference on Localization and GNSS (ICL-GNSS 2012).

[18] Del Peral-Rosado J.A., Barreto-Arboleda M.A., Zanier F., Seco-Granados G., Lopez-Salcedo J.A. Performance limits of V2I ranging localization with LTE networks. Proceedings of the 2017 14th Workshop on Positioning, Navigation and Communications (WPNC 2017).

[19] Knutti F., Sabathy M., Driusso M., Mathis H., Marshall C. Positioning Using LTE Signals. Proceedings of the European Navigation Conference.

[20] Shamaei K., Khalife J., Kassas Z.M. Performance characterization of positioning in LTE systems. Proceedings of the 29th International Technical Meeting of the Satellite Division of the Institute of Navigation (ION GNSS 2016).

[21] (2017). ETSI TS 136 211. Physical Channels and Modulation. Standard 3GPP TS 36.211, Rel. 14. https://www.etsi.org/deliver/etsi_ts/136200_136299/136211/14.02.00_60/ts_136211v140200p.pdf.

[22] Mensing C., Plass S. Positioning Algorithms for Cellular Networks Using TDOA. Proceedings of the 2006 IEEE International Conference on Acoustics Speech and Signal Processing Proceedings.

[23] (2017). ETSI TS 136 355. LTE Positioning Protocol (LPP). Standard 3GPP TS 36.355, Rel. 14. https://www.etsi.org/deliver/etsi_ts/136300_136399/136355/14.02.00_60/ts_136355v140200p.pdf.

[24] Del Peral-Rosado J.A., Parro-Jiménez J.M., López-Salcedo J.A., Seco-Granados G., Crosta P., Zanier F., Crisci M. Comparative results analysis on positioning with real LTE signals and low-cost hardware platforms. Proceedings of the 2014 7th ESA Workshop on Satellite Navigation Technologies and European Workshop on GNSS Signals and Signal Processing (NAVITEC).

[25] (2019). Study on NR Positioning Support. https://portal.3gpp.org/desktopmodules/Specifications/SpecificationDetails.aspx?specificationId=3501.

[26] Koivisto M., Costa M., Werner J., Heiska K., Talvitie J., Leppänen K., Koivunen V., Valkama M. (2017). Joint Device Positioning and Clock Synchronization in 5G Ultra-Dense Networks. IEEE Trans. Wirel. Commun..

[27] Del Peral-Rosado J.A., Raulefs R., López-Salcedo J.A., Seco-Granados G. (2018). Survey of Cellular Mobile Radio Localization Methods: From 1G to 5G. IEEE Commun. Surv. Tutor..

[28] Wymeersch H., Seco-Granados G., Destino G., Dardari D., Tufvesson F. (2017). 5G mmWave Positioning for Vehicular Networks. IEEE Wirel. Commun..

[29] Kaune R., Horst J., Koch W. Accuracy analysis for TDOA localization in sensor networks. Proceedings of the 14th International Conference on Information Fusion.

[30] Chan Y.T., Ho K.C. (1994). A simple and efficient estimator for hyperbolic location. IEEE Trans. Signal Process..

[31] Chang C., Sahai A. Estimation bounds for localization. Proceedings of the 2004 First Annual IEEE Communications Society Conference on Sensor and Ad Hoc Communications and Networks (IEEE SECON 2004).

[32] Sivers M., Fokin G. (2015). LTE Positioning Accuracy Performance Evaluation. Internet of Things, Smart Spaces, and Next Generation Networks and Systems. Internet of Things, Smart Spaces, and Next Generation Networks and Systems.

[33] Han Y., Shen Y., Zhang X.P., Win M.Z., Meng H. (2016). Performance limits and geometric properties of array localization. IEEE Trans. Inform. Theory.

[34] Chen C.S., Lin J.M., Liu W.H., Chi C.L. (2011). Dilution of position calculation for MS location improvement in wireless communication systems. J. Netw..

[35] Yong Y., Lingjuan M. GDOP results in all-in-view positioning and in four optimum satellites positioning with GPS PRN codes ranging. Proceedings of the PLANS 2004. Position Location and Navigation Symposium (IEEE Cat. No.04CH37556).

[36] Le D.V., Kamminga J.W., Scholten H., Havinga P.J. Error bounds for localization with noise diversity. Proceedings of the 12th Annual International Conference on Distributed Computing in Sensor Systems (DCOSS 2016).

[37] Chen C.S., Chiu Y.J., Lee C.T., Lin J.M. (2013). Calculation of weighted geometric dilution of precision. J. Appl. Math..

[38] Del Peral-Rosado J.A., López-Salcedo J.A., Kim S., Seco-Granados G. Feasibility study of 5G-based localization for assisted driving. Proceedings of the 2016 International Conference on Localization and GNSS (ICL-GNSS 2016).

[39] Li W., Hu X., Gao J., Zhao L., Jiang T. (2020). Measurements and Analysis of Propagation Channels in Vehicle-to-Infrastructure Scenarios. IEEE Trans. Veh. Technol..

[40] Aygun B., Boban M., Vilela J.P., Wyglinski A.M. Geometry-based propagation modeling and simulation of vehicle-to-infrastructure links. Proceedings of the 2016 IEEE 83rd Vehicular Technology Conference.

[41] Yi G., Shengchu W., Yupeng Z., Feng L., Lin Z. Exploring LTE-V link level performance by geometry enhanced winner II channel model. Proceedings of the International Conference on Information Networking.

[42] Möller A., Nuckelt J., Rose D.M., Kürner T. Physical layer performance comparison of LTE and IEEE 802.11p for vehicular communication in an urban NLOS scenario. Proceedings of the 2014 IEEE 80th Vehicular Technology Conference.

[43] Gozalvez J., Sepulcre M., Bauza R. (2012). IEEE 802.11p vehicle to infrastructure communications in urban environments. IEEE Commun. Mag..

[44] (2010). IEEE Standard for Information Technology—Local and Metropolitan Area Networks—Specific Requirements—Part 11: Wireless LAN Medium Access Control (MAC) and Physical Layer (PHY) Specifications Amendment 6: Wireless Access in Vehicular Environments.

[45] Halili R., Weyn M., Berkvens R., Barolli L., Hellinckx P., Natwichai J. (2020). Localization Accuracy Performance Comparison Between LTE-V and IEEE 802.11p. Advances on P2P, Parallel, Grid, Cloud and Internet Computing.

[46] IEEE Standard 802.11 (2012). Part 11: Wireless LAN Medium Access Control (MAC) and Physical Layer (PHY) Specifications.

[47] (2019). ETSI TS 136 101. E-UTRA; User Equipment (UE) Radio Transmission and Reception. Standard 3GPPTS 36.101, Rel 14. https://www.etsi.org/deliver/etsi_ts/136100_136199/136101/14.03.00_60/ts_136101v140300p.pdf.

[48] Cohda Wireless (2015). Cohda Mobility MK5 Module Datasheet, V1.2.0.

[49] 5GAA (2019). V2X functional and performance test report; test procedures and results, 5G Automot. Assoc., Munich, Germany, 5GAA White Paper. 3GPP.

[50] Meinilä J., Kyösti P., Jämsä T., Hentilä L. (2010). WINNER II Channel Models. Radio Technol. Concept. IMT Adv..

[51] (2009). Further Advancements for E-UTRA Physical Layer Aspects. https://portal.3gpp.org/desktopmodules/Specifications/SpecificationDetails.aspx?specificationId=2493.

[52] Yan L., Lu Y., Zhang Y. (2017). An improved NLOS identification and mitigation approach for target tracking in wireless sensor networks. IEEE Access.

[53] Kay S.M. (1993). Fundamentals of Statistical Signal Processing: Estimation Theory.

[54] Bensky A. (2016). Wireless Positioning Technologies and Applications.

